# Inequitable nutritional impact of India’s first COVID-19 lockdown: a national cross-sectional analysis

**DOI:** 10.1186/s44263-026-00326-2

**Published:** 2026-10-01

**Authors:** Anushka Reddy Marri, Madolyn R. Dauphinais, Leonardo Martinez, Meagan Karoly, Finn McQuaid, Pranay Sinha

**Affiliations:** 1 Department of Global Health, Boston University School of Public Health, Boston, MA USA; 2 Section of Infectious Diseases, Department of Medicine, Boston Medical Center and Boston University Chobanian & Avedisian School of Medicine, Boston, MA USA; 3 Department of Epidemiology, Boston University School of Public Health, Boston, MA USA; 4 Department of Infectious Disease Epidemiology, London School of Hygiene and Tropical Medicine, London, UK

**Keywords:** COVID-19 lockdown, Nutritional disparities, India, Malnutrition, National family health survey

## Abstract

**Background:**

India’s first nationwide COVID-19 lockdown in March 2020 disrupted food systems, healthcare access, and livelihoods, raising concerns about its impact on nutritional outcomes. While the effects of the pandemic on food security have been widely documented, national-level evidence on nutritional status, particularly across economic strata, remains limited.

**Methods:**

We analyzed data from India’s fifth National Family Health Survey (NFHS-5), a national household survey conducted between 2019 and 2021, during which the COVID-19 lockdown occurred. We assessed underweight status (Body Mass Index (BMI) < 18.5 kg/m²) in adults, wasting (weight-for-height z-score < -2) in children under five, and anemia in men, women, and children under five. Outcomes were analyzed by wealth quintile (Q1-Q5). Adjusted prevalences were estimated by marginal standardization. Survey-weighted multivariable logistic regression models were used to examine associations between lockdown period and outcome prevalence, and concentration indices and curves were applied to assess changes in economic disparities.

**Results:**

After adjustment, underweight prevalence among men increased only in the near-poor second quintile (adjusted Odds Ratio (aOR) 1.25, 95% Confidence Interval (CI) 1.12–1.40) and declined in the wealthiest, while among women it declined in the wealthier quintiles and overall and was largely unchanged among the poorest. Wasting declined across all wealth quintiles in children, with smaller reductions among the poorest. Anemia declined across all groups. All outcomes remained concentrated in the poorest quintiles in both periods. Concentration indices and curves confirmed widening disparities in underweight and wasting, with smaller or less consistent shifts for anemia.

**Conclusions:**

Following India’s first COVID-19 lockdown, undernutrition improved for most groups, but these gains were inequitably distributed: improvements were concentrated among wealthier households while the poorest improved least, and near-poor men experienced a rise in underweight. Anemia declined across all groups but remained highest in the lower wealth quintiles. These estimates should be interpreted with caution, as the cross-sectional design precludes causal inference and higher mortality among the most vulnerable during the lockdown may have rendered post-lockdown estimates conservative. These findings point to the need for equity-focused interventions and resilient nutrition systems to protect at-risk populations during and after crises.

**Supplementary Information:**

The online version contains supplementary material available at https://doi.org/10.1186/s44263-026-00326-2.

## Background

The COVID-19 pandemic posed unprecedented challenges to public health systems globally, with low- and middle-income countries like India bearing a disproportionate burden [1, 2]. As the most populous low- and middle-income country (LMIC) with substantial economic and geographic diversity, India serves as a critical case for understanding how public health crises may affect vulnerable populations. Even prior to the pandemic, India had borne high levels of undernutrition, wasting, and anemia, particularly among impoverished segments of the population [3, 4]. In March 2020, a strict nationwide lockdown was implemented, further disrupting food systems, healthcare access, and economic livelihoods [5, 6]. These disruptions heightened concerns about worsening nutritional outcomes, particularly among economically vulnerable groups [7, 8].

Although the pandemic’s effects on food security are well documented, evidence on changes in nutritional status is limited [5, 6, 8]. To address that gap, we analyzed data from the fifth round of the National Family Health Survey (NFHS-5), a nationally representative survey, to assess changes in the prevalence of key nutritional indicators (underweight status, anemia, and wasting) in men, women, and children before and after the lockdown. We also examined how these impacts differed in magnitude across wealth quintiles to understand the effects on existing nutritional disparities. Our study uses the phased rollout of NFHS-5, which allows a rare opportunity to compare nutrition indicators before and after the lockdown. Specifically, we asked: Did the COVID-19 lockdown exacerbate nutritional disparities across wealth groups in India? Understanding such effects is critical to shaping policy responses that prioritize equity and mitigate disproportionate disease burden during and after future public health or economic crises [7].

## Methods

NFHS is India’s iteration of the Demographic and Health Surveys (DHS) program, a series of household surveys conducted using standardized questionnaires, biomarker protocols, and field procedures across countries and survey rounds [9]. Data collection follows a stratified, two-stage cluster sampling design, with census enumeration areas selected as primary sampling units (PSUs) and households sampled systematically within each PSU [9]. Separate survey modules are administered to households, men, women, and children under five years. Although NFHS-5 fieldwork was conducted in phases across different regions, leading to residual regional differences, the sampling design and weighting procedures were implemented consistently across phases and are intended to support national-level comparability [9].

**Fig. 1:**
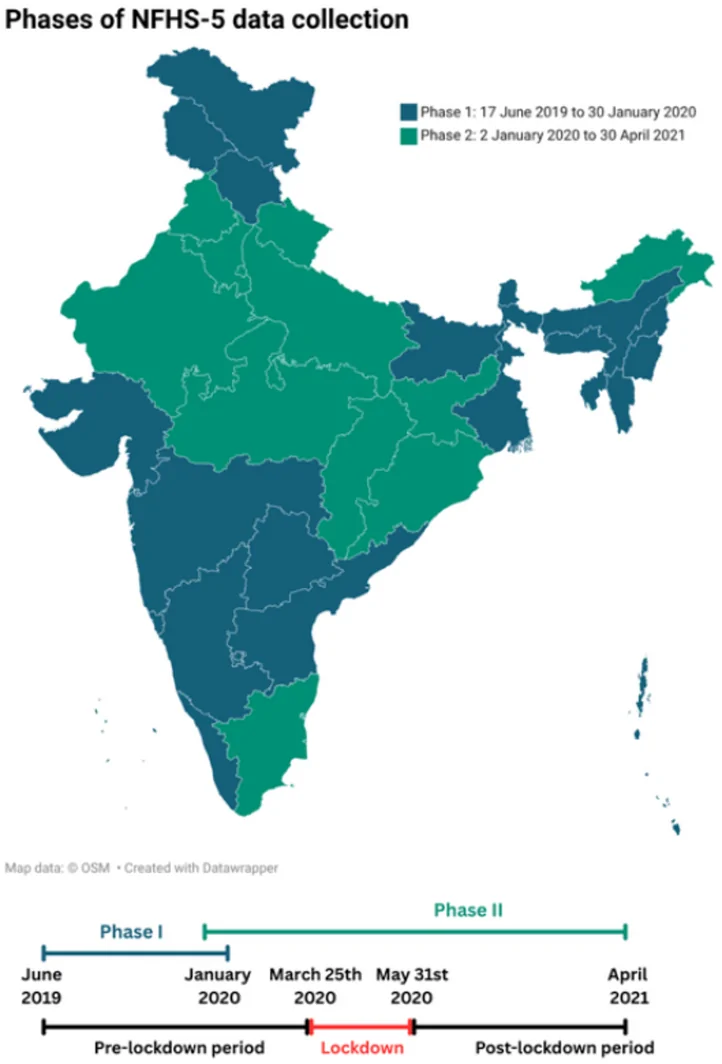
NFHS-5 survey phases and analytic lockdown periods. Legend: This figure displays the Indian states and union territories covered in Phase 1 (June 2019–January 2020) and Phase 2 (January 2020–April 2021) of the National Family Health Survey (NFHS-5). The timeline below the map illustrates the overlap between survey fieldwork and India’s first nationwide COVID-19 lockdown (March 25–May 31, 2020). Regions surveyed exclusively during Phase 1 contributed only to the pre-lockdown analytic sample. Regions surveyed during Phase 2 contributed data to either the pre-lockdown or post-lockdown period depending on the month of interview. The pre- and post-lockdown samples represent independent cross-sectional observations drawn from the same national source population; no individual participants were followed over time. The map was created using Datawrapper

NFHS-5 covered 636,699 households across all Indian states and union territories in two phases, covering different regions: June 2019–January 2020 and January 2020–April 2021 [10]. India’s first nationwide COVID-19 lockdown was implemented between March 25 and May 31, 2020 [11]. We defined “pre-lockdown” as June 2019 to March 2020 (included all of phase one and the early months of phase two) and “post-lockdown” as June 2020 to April 2021 (included data from phase two only). No data were collected during the lockdown period. Because the pre- and post-lockdown periods drew observations from different combinations of NFHS-5 survey phases, the geographic composition of the samples differed. The overlap between the NFHS-5 survey phases and the lockdown periods used for this analysis is depicted in Fig. 1.

### Study population and participant selection

Of the 636,699 households surveyed in NFHS-5, nutritional and biomarker measurements were collected in specific subsamples, as part of their standard sampling framework. Therefore, our analytical sample included adult men aged 15–54 years, adult women aged 15–49 years, and children under five years (0–59 months) of age.

Individuals were included if they had non-missing data for the relevant outcome. Exclusions were outcome-specific and applied at the individual level. As a result, analytic sample sizes vary by outcome. Due to the cross-sectional design of NFHS-5, individuals surveyed before and after the lockdown represent independent samples drawn from the same national source population, rather than the same individuals followed over time.

### Nutritional outcome measures

Nutritional indicators were based on standard anthropometric and hemoglobin measurements collected by trained NFHS survey staff.

Underweight status in adults: For adult men (aged 15–54 years) and women (aged 15–49 years), underweight status was defined as body mass index (BMI) less than 18.5 kg/m² [3]. We used the BMI variable provided in the NFHS-5 recode datasets, which is calculated by the survey program using measured height and weight.

Wasting in children: For children under 5 years of age, wasting was defined as weight-for-height z-score [WHZ] less than − 2 standard deviations, using World Health Organization (WHO) growth standards [3]. We used the WHO-standardized WHZ values provided in the dataset.

Anemia: Anemia status was determined using hemoglobin concentrations, adjusted for altitude for all populations and additionally for smoking status among adults, according to WHO standards [4]. Among adult men aged 15–54 years, mild anemia was defined as hemoglobin 11.0–12.9 g per deciliter (g/dL), moderate anemia as 8.0–10.9 g/dL, and severe anemia as < 8.0 g/dL. Among adult women aged 15–49 years, mild anemia was defined as hemoglobin 11.0–11.9 g/dL in non-pregnant women or 10.0–10.9 g/dL in pregnant women, moderate anemia as 8.0–10.9 g/dL in non-pregnant women or 7.0–9.9 g/dL in pregnant women, and severe anemia as < 8.0 g/dL in non-pregnant women or < 7.0 g/dL in pregnant women. Among children under 5 years of age, mild anemia was defined as hemoglobin 10.0–10.9 g/dL, moderate anemia as 7.0–9.9 g/dL, and severe anemia as < 7.0 g/dL. For all populations, “any anemia” was defined as the presence of mild, moderate, or severe anemia, collapsed into a single binary outcome.

### Wealth quintiles

Household economic status is measured as wealth index, a composite indicator constructed using household asset ownership, housing characteristics, and access to services. The wealth index adjusts for urban-rural differences through a multi-step principal components analysis and produces a nationally comparable combined wealth score. We used the pre-defined wealth quintiles provided in the NFHS-5 dataset, which assign each household member to one of the five quintiles based on the national distribution of the combined wealth index score. We use Q1-Q5 to denote wealth quintiles one through five, with Q1 representing the poorest households and Q5 the wealthiest.

### Statistical analysis

All analyses were conducted separately for men, women, and children under five years of age. Population-specific survey weights were applied separately for men, women, and children under five years of age to account for the complex sampling design and unequal probabilities of selection and non-response. Weighted analyses were used to estimate the prevalence of underweight status, wasting, and anemia in the pre- and post-lockdown periods, overall and stratified by wealth quintile. Because the pre- and post-lockdown samples differed in geographic and sociodemographic composition, the main analysis reports covariate-adjusted prevalence estimates. These were obtained by marginal standardization, adjusting for age, caste, and religion, so that prevalences for both periods reflect a common population composition (that of the pooled analytic sample) [12]. The statistical significance of pre- versus post-lockdown differences was determined from the survey-weighted logistic regression models described below.

To examine relative changes in nutritional outcomes, survey-weighted logistic regression models were fitted with post-lockdown status as the primary exposure. Models were adjusted for age, caste, and religion, and adjusted odds ratios (aOR) with 95% confidence intervals (95% CI) were reported. Models were stratified by wealth quintile to assess differential effects across economic groups.

Economic inequality in nutritional outcomes was assessed using survey-weighted concentration indices and concentration curves [13]. A negative concentration index indicates that adverse outcomes are more prevalent in lower wealth quintiles, while a positive value reflects a skew toward higher quintiles. An index of *zero* represents perfect equity. In concentration curves, this is shown by the *45-degree line of equity* (dotted line). Concave curves indicate concentration among poorer populations, while convex curves reflect concentration among richer populations [13].

As a sensitivity analysis, we restricted analyses to the 13 states and union territories with observations available in both the pre- and post-lockdown periods to assess whether differences in state sampling across phases influenced the findings. Chandigarh, although classified within Phase II of NFHS-5, was excluded from these analyses because no pre-lockdown observations were available. Adjusted prevalence estimates and aORs were recalculated separately for men, women, and children under five years of age overall and by wealth quintile using the same analytical approach as the primary analyses.

All data management and statistical analyses were conducted in R (version 4.5.1) [14]. All analysis code is available in a public repository (see Availability of data and materials) [15].

## Results

The unweighted analytical sample in the pre-lockdown period included 69,844 men, 496,427 women, and 153,886 children under five, and 31,995 men, 227,688 women, and 70,332 children under five in the post-lockdown period. Table 1 summarizes the sociodemographic characteristics of pre- and post-lockdown analytical samples.

**Table 1:** Sociodemographic characteristics of pre- and post-lockdown analytical samples by population group, NFHS-5 (2019–2021)

Lockdown period (*N*)	Men (15–54 years)		Women (15–49 years)		Children under five years	
	Pre (69844)	Post (31995)	Pre (496427)	Post (227688)	Pre (153886)	Post (70332)
Age (mean, SD) years (adults); months (children under five)	32.3 (11.2)	31.8 (11.1)	30.5 (10)	30.1 (9.9)	29.7 (17.4) (months)	29.2 (17.5) (months)
Religion (%)						
Hindu	73%	81%	80%	84%	78%	82%
Muslim	14%	8%	15%	11%	18%	13%
Other	11%	9%	5%	5%	4%	5%
Caste (%)						
Scheduled Caste/ Scheduled Tribe	36%	38%	31%	35%	33%	38%
Other Backward Caste (OBC)	39%	42%	43%	47%	44%	47%
Other	22%	19%	24%	17%	21%	15%
Rural residence (%)	68%	70%	67%	68%	73%	74%

Legend: N = unweighted analytical sample size. Percentages are weighted using population-specific survey weights. Percentages may not sum to 100% because of missing values and rounding

After adjusting for age, caste, and religion, underweight prevalence among men was 14.4% pre-lockdown and 14.6% post-lockdown. Among women, it decreased from 19.0% to 17.5%, and wasting prevalence among children under five declined from 20.4% to 16.3%. Anemia prevalence decreased in all groups (Table 2).

**Table 2:** Weighted frequencies and adjusted prevalence (%) of undernutrition, wasting, and anemia among men, women, and children under five years, pre- and post-lockdown

Nutritional indicator	Pre-lockdown	Post-lockdown
Men (15–54 years)		
Total sample for underweight (N)	66,083/70,719	27,906/30,291
Underweight prevalence (n, %)	9356 (14.4)	4148 (14.6)
Total sample for anemia (N)	65,587/70,719	27,288/30,291
Anemia prevalence (n, %)	16,907 (25.3)	5735 (20.9)
**Women (15–49 years)**		
Total sample for underweight (N)	499,233/518,880	193,005/205,235
Underweight prevalence (n, %)	92,779 (19.0)	34,471 (17.5)
Total sample for anemia (N)	493,440/518,880	188,593/205,235
Anemia prevalence (n, %)	288,894 (58.4)	100,083 (52.9)
**Children under five years**		
Total sample for wasting (N)	141,945/159,318	55,874/62,915
Wasting prevalence (n, %)	28,847 (20.4)	9178 (16.3)
Total sample for anemia (N)	130,509/159,318	48,753/62,915
Anemia prevalence (n, %)	90,741 (69.7)	31,210 (63.8)

Legend: N represents survey-weighted analytic sample size for each outcome; n represents survey-weighted number meeting the outcome definition; Prevalence values are adjusted for age, caste, and religion using marginal standardization. Significance of pre- versus post-lockdown differences is shown by the adjusted odds ratios (Fig. 2)

**Fig. 2:**
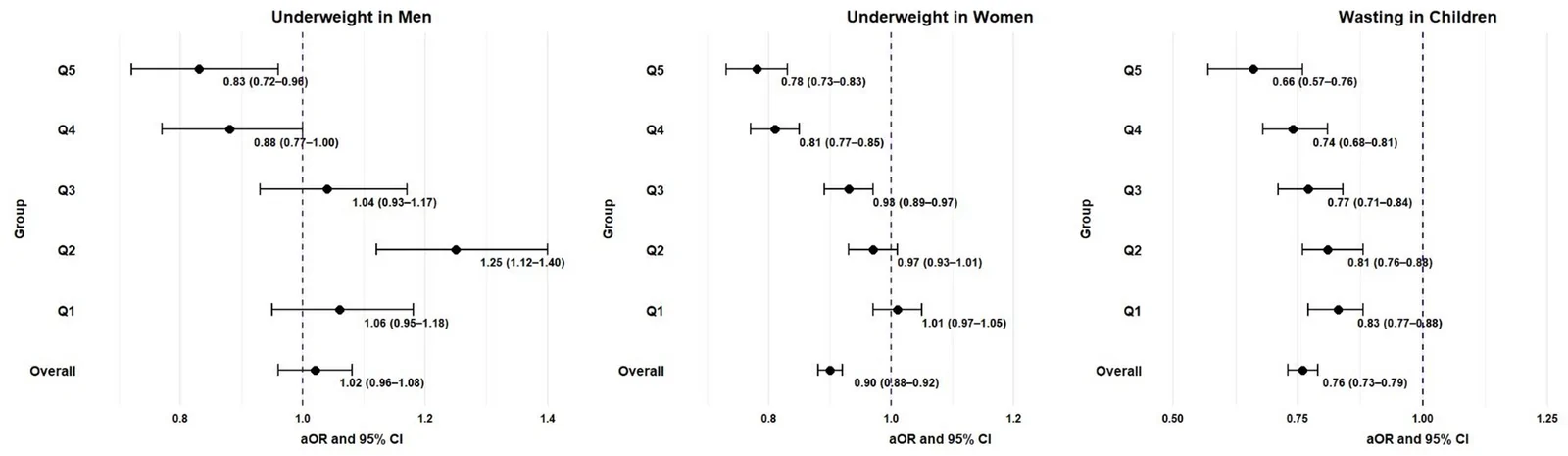
Adjusted odds ratios for nutritional outcomes post- versus pre-lockdown. Legend: Forest plots display survey-weighted adjusted odds ratios (aORs) and 95% confidence intervals (CIs) comparing post-lockdown versus pre-lockdown periods across five wealth quintiles (Q1–Q5) and the overall dataset. Outcomes include underweight in men and women (BMI < 18.5 kg/m²), wasting in children under five (weight-for-height z-score < − 2 SD), and anemia in men, women, and children under five (hemoglobin below WHO-defined thresholds). Models were adjusted for age, caste, and religion. The dashed vertical line indicates the null value (aOR = 1.0). aORs greater than 1.0 indicate higher odds of the outcome in the post-lockdown period, whereas aORs less than 1.0 indicate lower odds in the post-lockdown period

The changes in underweight status and wasting across wealth quintiles were non-uniform (Table 3). Specifically, the prevalence of underweight among adult men increased post-lockdown in the poorest quintiles [Q1 + 1.0%, Q2: +3.0%] and decreased in the wealthier quintiles [Q4 -1.3%, Q5: -1.4% ]. Among women, changes in the poorest quintiles were minimal (Q1 + 0.2%, Q2 -0.6%), while decreases were larger in the wealthier quintiles (Q4 -2.5%, Q5 -2.3%), indicating that the post-lockdown decline in women’s underweight was concentrated in higher wealth groups. Among children under five, wasting prevalence declined across all quintiles with smaller reductions in quintiles one and two [-3.2% in both] compared to larger reductions in quintiles four and five [-4.3% and − 5.4%]. Anemia prevalence decreased across wealth quintiles among all populations, though reductions were smallest in the richest quintile [men: -3.7%, women: -1.7%, children under five: -2.5%]. Despite these improvements, anemia, similar to underweight and wasting, remained most concentrated in the poorest wealth quintiles.

**Table 3:** Survey-weighted adjusted prevalence (%) by wealth quintile before and after lockdown

Variable	Population	Lockdown Status	Quintile 1 (%)	Quintile 2 (%)	Quintile 3 (%)	Quintile 4 (%)	Quintile 5 (%)
Underweight	Men	Pre-lockdown	20.1	16.0	14.3	12.7	9.5
		Post-lockdown	21.1	19.0	14.8	11.4	8.1
	Women	Pre-lockdown	25.8	21.6	18.8	16.5	12.8
		Post-lockdown	26.0	21.0	17.8	14.0	10.5
Wasting	Children	Pre-lockdown	23.3	20.8	19.6	19.4	17.8
		Post-lockdown	20.1	17.6	15.9	15.1	12.4
Anemia	Men	Pre-lockdown	31.4	27.4	25.4	22.8	20.6
		Post-lockdown	26.0	23.2	20.7	18.4	16.9
	Women	Pre-lockdown	64.5	61.1	58.4	55.7	52.7
		Post-lockdown	57.7	53.4	51.9	51.0	51.0
	Children	Pre-lockdown	74.1	70.3	69.2	68.1	64.9
		Post-lockdown	67.8	64.3	62.6	61.1	62.4

Legend: Pre- and post-lockdown survey-weighted prevalence estimates adjusted for age, caste, and religion using marginal standardization are shown for underweight status in adults, wasting in children under five, and anemia in all populations, stratified by wealth quintile (Q1 poorest to Q5 wealthiest)

Multivariable survey-weighted logistic regression models largely reinforced these patterns (Fig. 2). Adjusted odds ratios compare post-lockdown to pre-lockdown periods within each wealth quintile. Following the lockdown, the odds of underweight status among adult men increased significantly only in the second quintile [aOR: 1.25, 95% CI: 1.12–1.40]; the increase in the poorest quintile was not statistically significant [Q1 aOR: 1.06, 95% CI: 0.95–1.18], and the odds declined significantly in the wealthiest quintile [Q5 aOR: 0.83, 95% CI: 0.72–0.96]. Among adult women, the odds of underweight status were unchanged in the poorest quintiles [Q1 aOR 1.01, 95% CI: 0.95–1.05], and declined significantly in the third through fifth quintiles [Q5 aOR 0.78, 95% CI: 0.73–0.83] and overall [aOR: 0.90, 95% CI: 0.88–0.92]. Among children under five, the odds of wasting declined post-lockdown across wealth quintiles, with smaller reductions observed in poorer quintiles [Q1 aOR: 0.83, 95% CI: 0.77–0.88; Q2 aOR: 0.81, 95% CI: 0.76–0.88] and larger reductions in the wealthy [Q5 aOR: 0.66, 95% CI: 0.57–0.76]. The odds of anemia declined across all wealth quintiles and populations, post-lockdown, though the magnitude of decline varied.

**Fig. 3:**
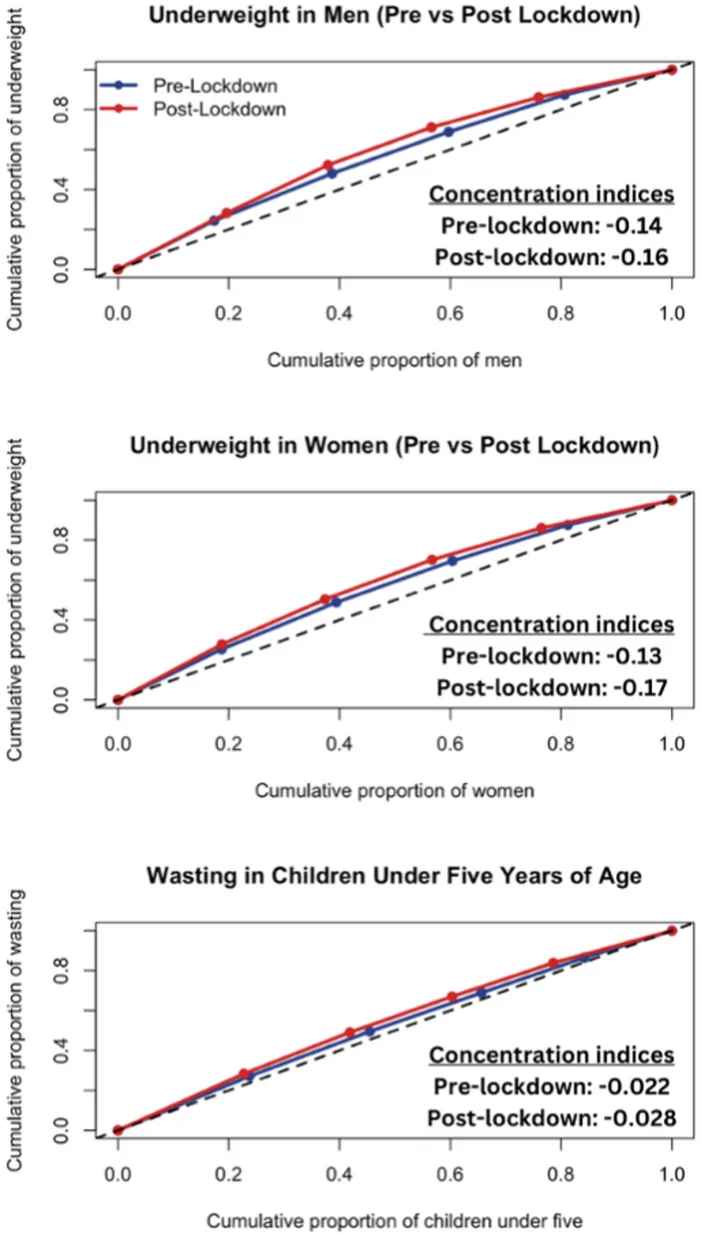
Concentration curves for underweight and wasting before and after lockdown. Pre- and post-lockdown concentration curves for underweight in men and women, and wasting in children under five, are shown from left to right. The dashed 45-degree line represents perfect equity. The blue curve indicates the pre-lockdown period, and the red curve indicates the post-lockdown period. Concentration indices are reported within each panel. Curves lying further below the line of equity indicate greater concentration of adverse outcomes among poorer wealth quintiles

Equity analyses using concentration indices showed increased concentration of underweight status and wasting among lower wealth quintiles after the lockdown. All concentration indices were negative, indicating that adverse nutritional outcomes were disproportionately concentrated among poorer wealth quintiles; movement farther from zero reflects increasing inequity, while movement toward zero indicates a reduction in inequity. Among adults, the index for underweight declined from − 0.13 to − 0.16 in men and − 0.13 to − 0.17 in women, indicating a growing burden among the poor. For children under five, the index for wasting also declined (from − 0.06 to − 0.10), suggesting a similar pattern. In contrast, trends for anemia were mixed and of smaller magnitude. Among men, the index shifted from − 0.09 to − 0.10, while among women it moved closer to zero (–0.04 to − 0.02), suggesting a modest reduction in inequity. Among children under five, it moved from − 0.022 to − 0.024, suggesting a slight shift toward greater concentration of anemia among poorer groups. These patterns were visually reinforced in the concentration curves (Fig. 3), which showed greater deviation from the line of equity post-lockdown, particularly for *underweight* status and wasting.

### Sensitivity analysis

Sensitivity analyses restricted to the 13 states and union territories contributing data to both analytic periods yielded results broadly consistent with the primary analyses. The increase in underweight odds among Q2 men was preserved (restricted aOR 1.26, 95% CI 1.09–1.45; primary aOR 1.25, 95% CI 1.12–1.40), and anemia declines were consistent across all populations and quintiles in both analyses.

Some attenuation was observed for wasting in children. Wasting improvements in Q1–Q3 children were no longer statistically significant in the restricted analysis (Q1: restricted aOR 0.93, 95% CI 0.86–1.02; primary aOR 0.83, 95% CI 0.77–0.88; Q2: restricted aOR 0.94, 95% CI 0.85–1.03; primary aOR 0.81, 95% CI 0.76–0.88; Q3: restricted aOR 0.95, 95% CI 0.86–1.05; primary aOR 0.77, 95% CI 0.71–0.84). Detailed results are provided in Supplementary material 1.

## Discussion

To our knowledge, this is the first national analysis of the COVID-19 lockdown’s unequal impact on nutrition across wealth groups in India. We observed divergent post-lockdown changes in nutritional outcomes, with overall national improvements masking widening disparities across wealth quintiles. The prevalence estimates were consistent with published NFHS-5 analyses reporting adult underweight, child wasting, and anemia across all populations [3, 4]. Underweight prevalence increased modestly among men [14.4% to 14.6%], but declined among women [19.0% to 17.5%], while wasting among children under five also declined overall [20.4% to 16.3%]. However, when stratified by household wealth, adult men in the poorest quintiles experienced increases in underweight prevalence, while higher wealth quintiles saw declines; among women, underweight was largely unchanged in the poorest quintiles and declined in wealthier groups. Similarly, reductions in wasting among children under five were smaller in lower wealth groups compared to larger declines among wealthier households. Over the past three NFHS rounds (NFHS-3 [2005–2006], NFHS-4 [2015–2016], and NFHS-5 [2019–2021]), India has experienced gradual declines in undernutrition indicators [3]. While the pre–post changes observed among women and children in our analysis broadly align with this longer-term downward trajectory, the reversal among men, who experienced a 0.2%-point increase in underweight prevalence, suggests a potential interruption of prior gains during the lockdown period.

While causal relationships cannot be established using cross-sectional data, the phased rollout of the NFHS-5 survey enabled a pre- and post-lockdown comparison using a large population-based national survey. It is important to note that the pre- and post-lockdown estimates reflect independent samples drawn from the same national source population, not longitudinal changes within individuals. The increase in underweight among near-poor men, together with the smaller nutritional gains observed among poorer groups relative to wealthier ones, likely reflect the combined effects of food insecurity, income loss, and reduced access to health and nutrition services during the lockdown. In a 2020 multi-country survey spanning 37 nations, 9 out of 10 households reported losing more than half of their income during the pandemic, and 8 in 10 faced difficulty purchasing food [16]. The disruptions to school feeding programs and community-based nutrition services likely contributed as well [17–20]. It is also likely that disparities deepened in the months following the lockdown, as disruptions to food access and health services persisted well beyond the initial lockdown [21]. The World Bank reported that by 2024, 343 million people across 74 countries were facing acute food insecurity, reflecting the lasting impacts of the COVID-19 pandemic, compounded by conflict and economic instability [22].

Notably, among men, Q2 households showed somewhat higher odds of underweight than Q1, which may reflect gaps in safety net coverage among near-poor households who fall above the Below Poverty Line eligibility thresholds and therefore may not have benefited from expanded food grain allocations under the Pradhan Mantri Garib Kalyan Anna Yojana during the lockdown [23]. This hypothesis was not consistent across all outcomes and warrants further investigation. Conversely, the improvements concentrated in wealthier quintiles may reflect their greater capacity to buffer the economic shock and their lesser exposure to lockdown-related disruptions in work and food access, consistent with evidence that poorer households in India bore a disproportionate economic burden during the pandemic [24]; however, this remains a hypothesis that cannot be tested directly with these data.

Due to the population size in India, even small shifts in nutrition metrics equate to large absolute human tolls: hundreds of thousands more wasted children and over ten million additional underweight adults. Together, these findings indicate that the nutritional impact of the lockdown was uneven and likely amplified pre-existing economic disparities.

Anemia prevalence declined overall [25.3% to 20.9% in men, 58.4% to 52.9% in women, 69.7% to 63.8% in children under five] but remained concentrated in poorer wealth quintiles in both periods. Although anemia prevalence improved overall, inequality in anemia among men increased slightly. Improvements were particularly limited among poorer children, suggesting persistent barriers to iron-rich diets and fortified foods [25]. Since nutritional anemia often develops gradually, our analysis of the immediate post-lockdown period may not have captured its full impact [26]. Longer-term data from Karnataka, for example, show that anemia prevalence doubled in rural children aged 6–12 years one year after the lockdown [17]. However, some factors, such as reduced exposure to intestinal parasites due to school closures and hygiene measures, as well as the ongoing Anemia Mukt Bharat initiative, may have contributed to observed reductions [27, 28].

These patterns should be interpreted in the context of the demographic disruptions accompanying the lockdown period. Mortality related to both the lockdown-associated hardship and to COVID-19 infection may have influenced post-lockdown estimates [24]. Although COVID-19 mortality data in India are incompletely ascertained, available evidence suggests that economically vulnerable populations may have faced elevated risk during the pandemic, raising the possibility of disproportionate mortality [24, 29]. This could have resulted in selective survival or displacement and consequently more conservative post-lockdown prevalence estimates. If nutritionally compromised individuals in poorer quintiles experienced higher mortality during the lockdown, they would be underrepresented in the post-lockdown sample, potentially biasing undernutrition prevalence downward among the poorest groups [29, 30]. As a result, our estimates of widening economic disparities may be conservative, and the true equity impact of the lockdown may be greater than reported.

Our analysis does have limitations. A key limitation of our analysis is the difference in geographic coverage between phases, which could introduce regional bias. However, the use of consistent definitions and standardized data collection protocols across states strengthens the internal validity of comparisons [10]. To evaluate the potential influence of geographic differences between analytic periods, we conducted a sensitivity analysis restricted to the 13 states and union territories represented in both periods. Attenuation of some findings in the restricted analysis likely reflects differences in geographic composition between the restricted and full analytic samples rather than loss of statistical power alone. The 13 overlapping states are not representative of the full national distribution, and restricting analyses to these states alters the population being described. Additionally, reduction in sample size in the restricted analysis may have contributed to attenuation of quintile-specific estimates, particularly among men where post-lockdown sample sizes were more limited. Taken together, the sensitivity analyses support the robustness of the primary findings while indicating that estimates for wasting improvements among children in lower wealth quintiles warrant cautious interpretation.

Without longitudinal follow-up, individual-level changes could not be assessed, limiting our ability to attribute observed differences directly to the lockdown. Moreover, anthropometric measures do not provide a full account of body composition or micronutrient deficiencies, which could have increased despite preserved BMI in upper wealth quintiles [31]. Other changes over time, such as state-level policies affecting food access, may also have shaped nutrition during the survey period. The NFHS data lack granular information on dietary intake or service utilization during the lockdown, restricting our ability to identify the proximate drivers of nutritional change.

Additionally, household wealth quintiles capture relative economic position but do not reflect other dimensions of socio-economic status such as education, occupation, or income, which may independently shape nutritional vulnerability [32]. Finally, although hemoglobin is the standard metric for assessing anemia, WHO cutoffs and field-based capillary measurements may overestimate prevalence in India, and hemoglobin alone cannot distinguish nutritional anemia from other common causes, warranting cautious interpretation [33].

Our findings indicate that protecting nutritionally vulnerable populations during crises demands more than stopgap relief: it requires durable safety nets, fortified food access, and uninterrupted nutrition services. Kerala’s experience with universal food baskets, combining free staple food distribution through the public distribution system, community kitchens, and targeted supplementary nutrition for women and children, demonstrates that equity-focused interventions can mitigate food insecurity during large-scale shocks [34]. Without such investments, future shocks, such as those from pandemics to extreme climate events, risk deepening cycles of malnutrition and poverty rather than breaking them [34].

## Conclusions

Following India’s first COVID-19 lockdown, underweight prevalence declined among women but rose modestly among men, and wasting declined among children under five. However, these improvements were inequitably distributed: gains were concentrated among wealthier households while the poorest groups improved least, and near-poor men experienced a rise in underweight. In contrast, anemia improved across all groups and more substantially among poorer quintiles, suggesting that targeted supplementation and hygiene interventions may have partially mitigated nutritional deficiencies during this period. These estimates should be interpreted in light of the study’s cross-sectional design, which precludes causal inference, and the potential for selective survival, whereby higher mortality among the most nutritionally vulnerable during the lockdown may have left a relatively better-off population in the post-lockdown samples and rendered our estimates conservative. These findings highlight the need for inclusive, equity-focused policy frameworks that sustain nutrition programs and safeguard vulnerable populations during future shocks.

## Supplementary Information

Below is the link to the electronic supplementary material.

Supplementary Material 1

## Data Availability

The NFHS-5 datasets analyzed in the current study are publicly available from the DHS Program (https://dhsprogram.com) upon registration. All analysis code is available at https://github.com/anrmarri/india-lockdown-nutrition-nfhs5-marri and archived at Zenodo (https://doi.org/10.5281/zenodo.21798750) [15]. The code was written in R (version 4.5.1) and is released under the MIT License; no restrictions apply to its use.

## Abbreviations

NFHS-5: National Family Health Survey – 5
DHS: Demographic and Health Survey
PSU: Primary sampling unit
Q1-Q5: Quintile 1–5
LMIC: low- and middle-income countries
BMI: body mass index
WHZ: weight-for-height z-score
WHO: World Health Organization
aOR: adjusted Odds ratio
95% CI: 95% Confidence Interval
g/dL: grams per deciliter
OBC: Other Backward Caste
