# Supplementary material for: Inequitable nutritional impact of India’s first COVID-19 lockdown: a national cross-sectional analysis
Source: BMC Glob Public Health. 2026 Oct 1;4:95. doi: 10.1186/s44263-026-00326-2 (PMC13629049; doi:10.1186/s44263-026-00326-2)
Supplement: Supplementary file 1 [file 44263_2026_326_MOESM1_ESM.pdf]

Supplementary Material 1

Sensitivity analysis restricted to NFHS-5 states and union territories with observations in both pre- and post-lockdown periods

Manuscript: Nutritional Impact of India's First COVID-19 Lockdown: Was It Equitable?

Overview

To evaluate the potential influence of geographic differences between survey phases on the primary findings, we conducted a sensitivity analysis restricted to the 13 states and union territories with observations available in both the pre- and post-lockdown periods of NFHS-5. Chandigarh, although classified within Phase II of NFHS-5, was excluded because no pre-lockdown observations were available. Weighted adjusted prevalence estimates and adjusted odds ratios (aORs) were recalculated separately for men, women, and children under five years of age, overall and by wealth quintile, using the same analytical approach as the primary analyses (survey-weighted logistic regression adjusted for age, caste, and religion, with adjusted prevalences obtained by marginal standardization). Tables S1–S6 present results for each outcome and population.

In each table, adjusted prevalence (%) and adjusted odds ratios (aOR) with 95% confidence intervals (CI) are shown for both the sensitivity analysis restricted to the 13 overlapping states and the primary analysis covering all states. Adjusted odds ratios compare post-lockdown to pre-lockdown periods within each wealth quintile, adjusted for age, caste, and religion. Wealth quintiles are denoted Q1 (poorest) through Q5 (wealthiest).

Table S1. Sensitivity analysis for underweight status among adult men (15–54 years), NFHS-5

	Overall	Q1	Q2	Q3	Q4	Q5
**Adjusted prevalence (%), 13 overlapping states**						
NFHS-5 pre-lockdown	15.3	22.7	17.4	15.3	12.3	10.0
NFHS-5 post-lockdown	15.6	22.6	21.0	16.3	12.2	8.4
**Adjusted prevalence (%), all states (primary analysis)**						
NFHS-5 pre-lockdown	14.4	20.1	16.0	14.3	12.7	9.5
NFHS-5 post-lockdown	14.6	21.1	19.0	14.8	11.4	8.1
**Adjusted odds ratio (95% CI), post- vs. pre-lockdown**						
13 overlapping states	1.04 (0.96–1.11)	1.02 (0.89–1.17)	1.26 (1.09–1.45)	1.08 (0.93–1.25)	0.97 (0.81–1.15)	0.84 (0.71–1.01)
All states (primary analysis)	1.02 (0.96–1.08)	1.06 (0.95–1.18)	1.25 (1.12–1.40)	1.04 (0.93–1.17)	0.88 (0.77–1.00)	0.83 (0.72–0.96)

Sensitivity analyses restricted to the 13 states and union territories surveyed in both NFHS-5 pre- and post-lockdown phases yielded findings broadly consistent with the primary analyses. Adjusted prevalence estimates in the restricted sample were similar to those observed nationally. The increased odds of underweight among men in the second wealth quintile were preserved in both analyses (restricted aOR 1.26, 95% CI 1.09–1.45; primary

aOR 1.25, 95% CI 1.12–1.40). Across both restricted and national analyses, poorer wealth quintiles continued to demonstrate substantially higher underweight prevalence compared with wealthier quintiles. These findings suggest that the observed associations were unlikely to be driven primarily by differences in states surveyed across NFHS-5 phases.

Table S2. Sensitivity analysis for anemia among adult men (15–54 years), NFHS-5

	Overall	Q1	Q2	Q3	Q4	Q5
**Adjusted prevalence (%), 13 overlapping states**						
NFHS-5 pre-lockdown	25.5	31.6	27.9	26.5	23.6	18.9
NFHS-5 post-lockdown	19.7	24.4	22.3	19.4	17.4	16.3
**Adjusted prevalence (%), all states (primary analysis)**						
NFHS-5 pre-lockdown	25.3	31.4	27.4	25.4	22.8	20.6
NFHS-5 post-lockdown	20.9	26.0	23.2	20.7	18.4	16.9
**Adjusted odds ratio (95% CI), post- vs. pre-lockdown**						
13 overlapping states	0.78 (0.72–0.83)	0.74 (0.66–0.84)	0.78 (0.69–0.87)	0.74 (0.65–0.84)	0.76 (0.66–0.87)	0.85 (0.74–0.97)
All states (primary analysis)	0.78 (0.74–0.82)	0.77 (0.70–0.85)	0.80 (0.72–0.89)	0.77 (0.69–0.85)	0.77 (0.69–0.86)	0.78 (0.70–0.88)

Sensitivity analyses for anemia among men yielded findings highly consistent with the primary analyses. Adjusted prevalence estimates in the restricted 13-state sample were similar to those observed nationally across wealth quintiles. From NFHS-5 pre- to post-lockdown periods, anemia prevalence declined overall and across all wealth quintiles in both restricted and national analyses, although poorer quintiles consistently

demonstrated higher anemia prevalence compared with wealthier groups. Adjusted odds ratios from the restricted analyses closely mirrored those from the national analyses, with similar relative reductions across wealth strata. These findings further support the robustness of the observed associations and suggest that differential state sampling across NFHS-5 phases was unlikely to substantially influence the results.

Table S3. Sensitivity analysis for underweight status among adult women (15–49 years), NFHS-5

	Overall	Q1	Q2	Q3	Q4	Q5
**Adjusted prevalence (%), 13 overlapping states**						
NFHS-5 pre-lockdown	18.9	26.5	22.0	18.9	15.7	12.3
NFHS-5 post-lockdown	17.9	26.0	21.3	18.4	14.6	10.9
**Adjusted prevalence (%), all states (primary analysis)**						
NFHS-5 pre-lockdown	19.0	25.8	21.6	18.8	16.5	12.8
NFHS-5 post-lockdown	17.5	26.0	21.0	17.8	14.0	10.5
**Adjusted odds ratio (95% CI), post- vs. pre-lockdown**						
13 overlapping states	0.93 (0.90, 0.95)	0.97 (0.93, 1.02)	0.96 (0.91, 1.01)	0.96 (0.91, 1.02)	0.91 (0.86, 0.97)	0.86 (0.81, 0.92)
All states (primary analysis)	0.90 (0.88–0.92)	1.01 (0.97–1.05)	0.97 (0.93–1.01)	0.93 (0.89–0.97)	0.81 (0.77–0.85)	0.78 (0.73–0.83)

Sensitivity analyses for underweight status among women yielded findings broadly consistent with the primary analyses. Adjusted prevalence estimates in the restricted 13-state sample were similar to those observed nationally, and poorer wealth quintiles consistently demonstrated substantially higher underweight prevalence compared with wealthier groups across all time periods. Adjusted odds ratios from the restricted

analyses were directionally similar to those from the national analyses but somewhat attenuated overall and in several quintiles. The overall decline in underweight among women remained statistically significant in the restricted analysis (restricted aOR 0.93, 95% CI 0.90–0.95; primary aOR 0.90, 95% CI 0.88–0.92). Overall, the socioeconomic patterning and temporal trends in underweight were unlikely to be explained primarily by differences in state sampling across phases.

Table S4. Sensitivity analysis for anemia among adult women (15–49 years), NFHS-5

	Overall	Q1	Q2	Q3	Q4	Q5
**Adjusted prevalence (%), 13 overlapping states**						
NFHS-5 pre-lockdown	56.9	62.4	58.3	56.9	54.8	52.9
NFHS-5 post-lockdown	52.9	57.5	53.2	52.1	51.1	51.1
**Adjusted prevalence (%), all states (primary analysis)**						
NFHS-5 pre-lockdown	58.4	64.5	61.1	58.4	55.7	52.7
NFHS-5 post-lockdown	52.9	57.7	53.4	51.9	51.0	51.0
**Adjusted odds ratio (95% CI), post- vs. pre-lockdown**						
13 overlapping states	0.85 (0.83, 0.87)	0.82 (0.78, 0.85)	0.81 (0.78, 0.85)	0.82 (0.79, 0.86)	0.86 (0.83, 0.90)	0.93 (0.89, 0.97)
All states (primary analysis)	0.80 (0.78–0.81)	0.75 (0.72–0.78)	0.73 (0.70–0.76)	0.77 (0.74–0.80)	0.83 (0.80–0.86)	0.94 (0.90–0.97)

Sensitivity analyses for anemia among women yielded findings generally consistent with the primary analyses, although overall anemia prevalence was somewhat lower in the restricted 13-state sample. Across all time periods, poorer wealth quintiles consistently demonstrated higher anemia prevalence compared with wealthier groups. From NFHS-5 pre- to post-lockdown periods, anemia prevalence declined overall and across most

wealth quintiles in both restricted and national analyses. Adjusted odds ratios from the restricted analyses were somewhat attenuated compared with national estimates but remained directionally consistent, with larger relative reductions generally observed in lower and middle wealth quintiles than in the wealthiest quintile. Overall, the socioeconomic patterning of anemia was unlikely to be explained primarily by differences in state sampling across phases.

Table S5. Sensitivity analysis for wasting among children under five years, NFHS-5

	Overall	Q1	Q2	Q3	Q4	Q5
**Adjusted prevalence (%), 13 overlapping states**						
NFHS-5 pre-lockdown	17.7	21.0	18.5	16.5	17.2	15.1
NFHS-5 post-lockdown	16.2	20.0	17.5	15.9	15.0	12.4
**Adjusted prevalence (%), all states (primary analysis)**						
NFHS-5 pre-lockdown	20.4	23.3	20.8	19.6	19.4	17.8
NFHS-5 post-lockdown	16.3	20.1	17.6	15.9	15.1	12.4
**Adjusted odds ratio (95% CI), post- vs. pre-lockdown**						
13 overlapping states	0.90 (0.86–0.94)	0.93 (0.86–1.02)	0.94 (0.85–1.03)	0.95 (0.86–1.05)	0.85 (0.76–0.95)	0.80 (0.71–0.89)
All states (primary analysis)	0.76 (0.73–0.79)	0.83 (0.77–0.88)	0.81 (0.76–0.88)	0.77 (0.71–0.84)	0.74 (0.68–0.81)	0.66 (0.57–0.76)

Sensitivity analyses for wasting among children under five years yielded findings broadly consistent with the primary analyses, although wasting prevalence was somewhat lower overall in the restricted 13-state sample. Wasting prevalence declined from NFHS-5 pre- to post-lockdown overall and across wealth quintiles in both analyses. Across all time periods, poorer wealth quintiles consistently demonstrated higher wasting prevalence

compared with wealthier groups. Adjusted odds ratios from the restricted analyses were attenuated relative to national estimates but remained directionally consistent, with inverse associations generally stronger in higher wealth quintiles. Wasting improvements in Q1 to Q3 children were no longer statistically significant in the restricted analysis (Q1: restricted aOR 0.93, 95% CI 0.86–1.02; primary aOR 0.83, 95% CI 0.77–0.88; Q2: restricted aOR 0.94, 95% CI 0.85–1.03; primary aOR 0.81, 95% CI 0.76–0.88; Q3: restricted aOR 0.95, 95% CI 0.86–1.05; primary aOR 0.77, 95% CI 0.71–0.84). Overall, the socioeconomic patterning and temporal trends were unlikely to be explained primarily by differences in state sampling across phases.

Table S6. Sensitivity analysis for anemia among children under five years, NFHS-5

	Overall	Q1	Q2	Q3	Q4	Q5
**Adjusted prevalence (%), 13 overlapping states**						
NFHS-5 pre-lockdown	72.4	75.5	73.2	71.8	71.8	68.7
NFHS-5 post-lockdown	63.7	67.5	64.1	62.5	61.1	62.6
**Adjusted prevalence (%), all states (primary analysis)**						
NFHS-5 pre-lockdown	69.7	74.1	70.3	69.2	68.1	64.9
NFHS-5 post-lockdown	63.8	67.8	64.3	62.6	61.1	62.4
**Adjusted odds ratio (95% CI), post- vs. pre-lockdown**						
13 overlapping states	0.66 (0.63–0.69)	0.66 (0.61–0.72)	0.64 (0.59–0.69)	0.64 (0.59–0.70)	0.60 (0.55–0.66)	0.75 (0.69–0.82)
All states (primary analysis)	0.76 (0.73–0.78)	0.73 (0.68–0.78)	0.75 (0.70–0.81)	0.73 (0.68–0.79)	0.73 (0.68–0.78)	0.89 (0.83–0.96)

Sensitivity analyses for anemia among children under five years yielded findings broadly consistent with the primary analyses, although anemia prevalence was somewhat higher overall in the restricted 13-state sample. Across all time periods, poorer wealth quintiles consistently demonstrated higher anemia prevalence compared with wealthier groups. Substantial reductions in anemia prevalence from NFHS-5 pre- to post-lockdown periods

were observed overall and across all wealth quintiles in both restricted and national analyses. Adjusted odds ratios from the restricted analyses demonstrated stronger inverse associations than the national analyses but remained directionally consistent across wealth strata. Overall, the socioeconomic patterning and temporal trends were unlikely to be explained primarily by differences in state sampling across NFHS-5 phases.

Summary

Across all six outcomes, sensitivity analyses restricted to the 13 overlapping states yielded findings directionally consistent with the primary analyses. The only notable qualification concerned reductions in wasting among children in the poorest wealth quintiles, which were no longer statistically significant in the restricted analysis. The 13 overlapping states are not representative of the full national distribution, and reduction in sample size in the restricted analysis may also have contributed to attenuation of quintile-specific estimates. Taken together, these analyses support the robustness of the primary findings, with the main qualification being that improvements in wasting among children in the poorest wealth quintiles were attenuated and not statistically significant in the restricted sample, and warrant cautious interpretation.
